# PD158 Optimizing The Management Of Patients With Mitral Regurgitation Beyond Technological Innovations: A Proposed Set Of Actions

**DOI:** 10.1017/S0266462324003891

**Published:** 2025-01-07

**Authors:** Paula Campelos, José Francisco Díaz, José López-Haldón, Luis Nombela-Franco, Eduardo Pinar, Ángel González-Pinto, Margarita Reina, Rubén Tarrío, Laura Vidal, Seila Lorenzo-Herrero, Jesús Cuervo, Marta Mengual, Paloma González

## Abstract

**Introduction:**

Mitral regurgitation (MR) is the most prevalent valvular heart disease worldwide and is frequently underdiagnosed and undertreated, resulting in a substantial healthcare burden. This project aimed to define an optimized patient journey, identifying specific unmet needs and pain points in the management of MR in Spain, and to propose a set of recommendations that can be implemented at a clinical level.

**Methods:**

Using the Population, Intervention, Comparator, and Outcomes search strategy, a pragmatic literature review was conducted to contextualize the comprehensive management of patients with MR in Spain. Subsequently, a Delphi panel consisting of two rounds of questionnaires was implemented. Unmet needs detected for MR management along the patient journey were validated by a panel of clinical experts incorporating different profiles. A battery of actions to improve the MR patient journey was also gathered (first round), which were then systematically reviewed and prioritized by the experts using hierarchical point allocation methods (second round) based on their relevance and feasibility within the National Health System.

**Results:**

A set of actions was proposed for the following core phases: detection-diagnosis, treatment-decision, treatment, and follow up. Actions for detection-diagnosis should be prioritized since boosting patient referral to specialized centers was considered crucial. Within the treatment-decision stage, experts emphasized strengthening healthcare services communication and training on risk stratification. For treatment, early referral to specialized centers was prioritized. Optimizing follow up required educating patients and relatives on adherence and self-care. Finally, experts supported a common pathway for heart valve diseases such as MR, tricuspid regurgitation, and aortic stenosis. Specifically, they concluded that optimization of tricuspid regurgitation management aligned with the actions proposed for MR.

**Conclusions:**

Altogether, unmet needs and critical aspects in each of the management steps of MR in Spain were detected and an array of potential actions was suggested by clinical experts. The evaluation of such actions resulted in a preliminary strategic plan that can help prioritize interventions and healthcare policies regarding the optimization of the healthcare journey for patients with MR (and other valvulopathies) in Spain.

